# Examining the effects of public participation on residents’ trust in local government: The mediating effect of perceived responsiveness

**DOI:** 10.1371/journal.pone.0323047

**Published:** 2025-05-12

**Authors:** Ahmed-Nor Mohamed Abdi, Ahmed Ibrahim Nageye, Hassan Omar Sabriye

**Affiliations:** Faculty of Economics and Management, Somali National University, Mogadishu, Somalia.; University of Pretoria, SOUTH AFRICA

## Abstract

Public participation has been widely acknowledged as a mechanism to enhance residents’ trust in governmental institutions. However, the specific pathways through which public participation influences trust via the perceived responsiveness of local government remain underexplored, particularly in post-war settings such as Mogadishu, Somalia. Hence, this study aimed to explore the impact of public participation on residents’ trust in local authorities in Mogadishu, Somalia, through the perceived responsiveness as a mediator in this relationship within the unique context of a post-war country. Data were collected between January 20 and April 30, 2024. This research employed a quantitative online survey to gather data from 446 inhabitants of Mogadishu City, Somalia. The subjects in the study were recruited using a convenient sampling technique. The proposed model was analyzed using covariance-based structural equation modeling (CB-SEM) in SmartPLS version 4 to examine the hypothesized relationships and mediating effects. The findings indicated that perceived public participation positively and significantly predicted both perceptions of responsiveness and residents’ trust in local authorities. The findings also revealed that perceptions of local government responsiveness were directly, positively, and significantly associated with residents’ trust in the city administration. The results ultimately indicated a positive partial mediation effect of perceived local government responsiveness on the association between public participation and residents’ trust in city administration. This study addresses a lacuna in the literature by exploring how perceived public participation influences residents’ trust in city authorities in a post-conflict setting, particularly in Somalia. This underscores the significance of perceived responsiveness as a mediator in enhancing residents’ trust, providing invaluable insights for policymakers and local government officials.

## Introduction

Effective democratic governance depends on establishing and maintaining high trust among residents in governmental organizations [1]. For many decades, political studies and public sector administration scientists have focused heavily on the issue of residents’ trust in the government [2–4]. Residents’ trust in public sector institutions has been a hot topic in the growing literature in the field of political science [5]. Moreover, residents who highly trust their state administration are more willing to engage in public events, respond to public sector agencies’ calls, comply with regulations, and follow their policies [6]. However, when residents no longer trust their government, they hesitate to collaborate with government institutions, which raises the costs and complexity of governing and may lead the government to be caught in the “Tacitus trap” [7]. Furthe rmore, Grimmelikhuijsen and Knies posited that assessing trust in a specific government agency, i.e., trust at the meso level, could contribute to addressing this paucity in the literature [3]. By gauging trust in public sector institutions, researchers can more precisely determine the specific institutional contexts associated with the predictors of trust in a particular institution. Zhao and Hu [8] argued that previous studies on residents’ trust in government institutions have mainly examined residents’ trust in the national government. Local authorities are closer to residents than the central government, and this proximity promotes increased interaction, resulting in significant structural differences between the two levels of government [9]. They further claimed that past studies have overlooked the predictors of residents’ trust in local authorities, where devolution of power has been advocated.

Despite the significance of residents’ trust in government institutions, past studies have reported a persistent decrease in this trust in industrialized and emerging countries worldwide [10,11]. However, Somalia is no exception to the trend of declining resident trust in government institutions, which can be ascribed to the oppressive military regime that ruled from 1969 to 1991, followed by subsequent state failure. In addition, Abdi and Hashi argued that persistent corruption, poor governance, and insufficient public service delivery undermined residents’ trust in public-sector agencies [12]. However, Somalia’s provisional federal constitution recognizes three levels of government, federal, state, and local, but responsibilities and power have yet to be devolved to local governments by the federal government in Somalia [13]. There is no genuine decentralization, so limited public participation in Somalia’s post-war governance system may reduce residents’ trust in city administration agencies. Against this backdrop, it is paramount to examine the predictors of residents’ trust in the local government in Mogadishu, Somalia.

Several past studies have examined how public participation predicts residents’ trust in public sector agencies. For instance, Han et al. in Nepal revealed that public participation predicts residents’ trust in the local government [9]. Similarly, past studies on public administration scholarship have reported that public participation is an essential antecedent of residents’ trust in government agencies [14–17]. Previous research has indicated that the local authority’s responsiveness is also an antecedent of trust in government [9,16,18,19]. Also, past research confirmed that public participation and responsiveness are associated [20].

Therefore, this study explores the impact of perceived public participation (PPP) on residents’ trust in local authorities (RTLG) in Mogadishu, Somalia. Moreover, it explores the mediating impact of perceived local government responsiveness (PRP) on this relationship. A plethora of earlier studies have explored the relationship between these constructs in separate investigations. However, the literature on public administration still needs a model that explains how perceived public participation influences residents’ trust in local government in Somalia, specifically through the mediating impact of perceived local government responsiveness. Furthermore, as far as the researchers is aware, no prior study has yet explored the mediating effect of perceived responsiveness on the association between perceptions of public participation and residents’ trust in local administration. Hence, to bridge this lacuna, this study proposes the following four research questions:

1. How do perceptions of public participation influence residents’ trust in the city government?
2. What is the impact of perceptions of public participation on the perceived responsiveness of city governments?
3. How does the perceived responsiveness of city governments influence residents’ trust in the city government?
4. Does the perceived responsiveness of the city government mediate the association between perceptions of public participation and residents’ trust in the city government?

This study gathered data from 446 inhabitants of Mogadishu City using an online quantitative survey to answer these four research questions. The findings offer invaluable insights to public administrators, local government policymakers, and future scholars investigating the evidence surrounding the study’s topic. Therefore, this study explores the impact of public participation on residents’ trust in local administration through the mediating effect of the perceived responsiveness of local government agencies.

## Literature review and hypotheses

### Public participation and residents’ trust in local government

Public participation is characterized as the public’s engagement in administrative responsibilities and decision making, facilitated through diverse channels available across several operational sectors, enabling public involvement in the decision-making process [21]. Furthermore, public participation in government is described as the involvement of citizens in the design or assessment of governmental aims, service benchmarks, policies, and outcomes through either direct or indirect means [22].

Public participation methods may take various participatory mechanisms, including public forums, advisory panels composed of residents, focus groups with citizens, dialogues involving enterprise stakeholders, and Chamber of Industry associations [9]. The functional sectors include community protection, transportation, land-use regulation, budgeting, planning, and other services typically delivered by the government [21]. The authors asserted that citizen participation in decision making involves active engagement in establishing objectives, formulating strategies, implementing policies, and evaluating. These studies emphasize the significance of involving residents at various stages of decision making, which can lead to policies that better reflect public needs and interests, thereby enhancing relationships of confidence and collaboration between the government and its citizens.

Scholars from diverse academic disciplines have proposed varying definitions of trust, reflecting its complex nature. In their seminal work, Rousseau et al. [23] defined trust as “a psychological state comprising the intention to accept vulnerability based upon positive expectations of the intentions or behavior of another.” Likewise, trust is a multifaceted psychological state [24]. This state encompasses the confidence, belief, readiness, assessment, and anticipation experienced by a person (the trustor) regarding a specific entity (the trustee). However, trust in government, despite the difficulty of defining it precisely, is a barometer of how the public feels about their government [25]. Moreover, trust is the anticipation that an individual or organization will conduct itself morally or competently [26]. He further described trust in public sector organizations as the extent to which the public believes the government and its representatives are capable, ethical, and responsive to their needs and choices. Trust in municipal administration institutions is grounded in residents’ confidence in municipal administration, reflecting the public’s logical evaluations, direct experiences, and idealized expectations regarding the functioning of local governance systems [27]. Similarly, trust in the public sector is defined as the residents’ trust in public sector agencies and representatives, signifying a person’s conviction or anticipation that the government will conform to accepted norms [28]. These scholarly conceptualizations suggest that trust in governmental institutions goes beyond simple abstract psychological states. It also serves as a dynamic measure of how well the government meets its constituents’ ethical and practical expectations. Researchers have frequently maintained that trust evaluations are multifaceted and incorporate a range of perceptions regarding government integrity, competence, and benevolence [29]. Moreover, the existing trust literature describes the three dimensions of trustworthiness: integrity, competence, and benevolence [3]. They defined competence as the degree to which the residents believe a public organization is effective, capable, professional, and trained. Integrity is the degree to which the public believes that a public sector organization is truthful, honest, and fulfils its promises. Finally, they defined benevolence as the degree to which a public believes that a public sector agency is concerned with the well-being of citizens and is driven to act in the public’s best interests. Furthermore, fundamentally, trust is a personal assessment of a relationship between an object (the one being trusted) and a person (the one giving the trust): B is trusted by A to do y [30]. Therefore, we would argue that this relational view of trust, where the act of trusting (B is trusted by A to do y) reflects a personal and evaluative process, helps explain why public participation can boost trust in local government.

Several previous studies have explored how public participation (PPP) influences residents’ trust in the government (RTLG). Noda surveyed 2,997 Tokyo residents in Japan and reported that public participation significantly influenced residents’ trust in local government leadership [31]. This study implies that when the public believes their views are respected and their voices heard, they will probably perceive their ownership of government agencies and trust in public institutions. Similarly, a survey study conducted by Han et al. [9] at three levels of local government in Nepal reported that public participation positively predicts residents’ trust in local administration. Moreover, Campbell conducted a vignette-based experiment in South Korea, which revealed that public participation enhances public trust in public sector organizations. [14] Also, Song et al.’s study in rural China revealed a positive relationship between government trust and public participation [32]. Goldfinch et al. [15] recently published a study from Japan that revealed a positive association between public participation and trust in city administrators. Turning our attention to the African continent, Arkorful et al. [17] surveyed four local Ghanaian governments. They found that public participation positively and significantly affected residents’ trust in local authorities in Ghana. Similarly, Beshi and Kaur [16] examined the effect of effective governance on residents’ trust in local administration in Ethiopia’s Bahir Dar local government. They found a significant association between public participation and residents’ trust in the local administration. This growing body of literature supports the claim that public participation enhances residents’ trust in government in various cultural and political contexts. Nonetheless, a study conducted by Holum [33] on local authorities in Norway found that trust in local politicians was not significantly related to citizen participation. This inconsistency in previous studies’ findings underscores the need for further research on this topic. Therefore, to explain the link between public participation and residents’ trust in local government, we used participatory theory [16]. According to Pateman’s theory of participatory democracy [34], public participation goes far beyond achieving better policy outcomes, a transformative process that deepens citizens’ trust in their government. Pateman argues that participation serves as an educational experience that creates a reciprocal relationship. As citizens become actively involved, they gain empowerment and build a foundation of trust that reinforces and legitimizes democratic institutions. In this view, trust is not an abstract quality handed down by the government but develops naturally through genuine engagement. When people feel their voices are heard and play a meaningful role in shaping public policy, their trust in the local government increases.

Despite the value of public participation in enhancing residents’ trust in city governments, the Somali context remains understudied and unexplored. Thus, this study suggests the ensuing hypothesis:

###  H1

*: Perceived public participation (PPP) is positively associated with residents’ trust in city government (RTLG).*

### Public participation and responsiveness

Different scholars have defined responsiveness in various ways. For instance, Yang and Pandey [35] defined responsiveness as the ability of institutions to accommodate residents’ preferences. Moreover, Koppell [36] defined responsiveness as an organization’s ability to express its constituents’ desires and needs directly. Koppell’s conceptualization posits that responsiveness extends beyond merely satisfying citizens’ preferences, encompassing the proactive expression and advocacy of public interest within decision-making frameworks. Furthermore, Perceived responsiveness is defined as public sector officials being attentive to and valuing residents’ views. Similarly, perceived responsiveness refers to the government’s propensity to react promptly and adequately to residents’ dissatisfaction and appeals to address their demands [38]. Their definition highlights that effective responsiveness involves recognizing public concerns and taking timely and appropriate actions to address them, emphasizing a proactive and results-oriented approach in public service provision. Similarly, responsiveness refers to the readiness and capacity of an institution, specifically the government, to address the needs and preferences of its residents [9]. Government responsiveness can be demonstrated by promptly addressing citizen inquiries, assisting those in need, and consistently delivering services in compliance with legal and regulatory standards [9]. In this study, responsiveness is defined as the capacity and readiness of local government institutions to listen to, prioritize, and address residents’ needs, preferences, and concerns.

A study by Chang and Meng [20] in 31 provinces of China, perceived public participation (PPP) and perceived responsiveness (PRP) were positively and significantly associated. Previous studies have argued that public participation can enhance the quality of information accessible to policymakers, resulting in more informed and responsive decision making [39]. Furthermore, a recent study asserted that engaging the inhabitants in the planning and execution process can guarantee that services and goods are delivered in a manner that genuinely addresses community demands and wants [40]. This study’s premise is that residents’ participation in the procedures of decision making facilitates the expression of residents’ concerns, needs, priorities, and preferences, ensuring their voices are acknowledged and enabling local authorities to respond effectively to community aspirations and requirements. Past studies on how public participation influences the responsiveness of local governments in general and specifically in Somali settings are scarce; therefore, this study hypothesizes that:

###  H2

*: Perceived public participation (PPP) is significantly and positively associated with perceived responsiveness (PRP).*

### Responsiveness and residents’ trust

A plethora of past studies explored the role of local authorities’ perceived responsiveness (PRP) in improving residents’ trust in city government (RTLG). Notably, Han et al. [9] conducted a survey study at three local governments in Nepal. Their study found that perceived local government responsiveness positively relates to residents’ trust in city governments. This study underscores the essential role that perceptions of responsiveness among the residents play in shaping the dwellers’ trust in their city authority agencies. Similarly, Nguyen et al. [18] a survey study of 529 residents in 11 cities of local governments in Vietnam showed that perceived responsiveness was significantly related to the inhabitants’ trust in the city’s electronic government services. The authors indicate that residents trust digital governance more when local governments respond quickly to public demands, preferences, and feedback. Responsiveness is crucial to bridging the gap between citizens and government, especially in online service delivery. Furthermore, Huang et al. [19] surveyed 4068 participants from China and reported that responsiveness has significantly shaped citizens’ trust in government institutions. This study makes the case that government responsiveness is crucial in shaping institutional trust in the public sector. Likewise, Beshi and Kaur’s [16] study of Ethiopia’s Bahir Dar local government, focusing on the Horn of Africa context, found a positive and significant association between inhabitants’ trust in city administration government and perceived local government responsiveness. While extensively documented in previous research, the linkage between city government responsiveness and residents’ trust in city government remains unexplored and understudied in Somalia. Thus, this study aims to address this notable gap in public administration literature by proposing the following hypotheses:

###  H3

*: Perceived local government responsiveness (PRP) is significantly and positively related to residents’ trust in city government (RTLG).*

### Perceived responsiveness as a mediator

Mediation research has become prominent in the social sciences due to recent theoretical developments that have clarified the prerequisites for recognizing, assessing, and interpreting mediating effects [41]. Moreover, it has become increasingly popular in social science research [42]. He further asserts that indirect relationship analysis (mediation analysis) determines the relationship that explains the association between an independent construct (X) and a dependent construct (Y) via a third construct (Z) that acts as a mediator. This approach holds significance for scholars, especially in areas with no prior mediating effects studies.

A recent study employed the perceived responsiveness of the government as a mediator in the relationship between government speed, usefulness of government responses, and citizens’ satisfaction in China [43]. However, to the best of our knowledge, we found that no prior study has explicitly modeled the mediating effect of the perceived responsiveness of local government (PRP) on the relationship between perceived public participation (PPP) and residents’ trust in local government (RTLG) based on the author’s comprehensive review of existing scholarly databases.

Nevertheless, myriad prior studies have confirmed that public participation predicts residents’ trust in local governments [9,15,17]. However, the studies assert that this is a partial relationship, and some effects indirectly occur via the perceived responsiveness of the local government. Earlier research has revealed a positive and significant association between public participation and responsiveness [20]. This study hypothesizes that perceived responsiveness mediates the association between public involvement and residents’ trust in the city government. Community participation in governmental decision making is essential for enhancing responsiveness and fostering trust among residents [9]. Engaging residents in decision-making processes, from goal setting to policy evaluation, provides governmental entities with valuable insights. This helps them understand community needs and preferences and tailor services accordingly. This can lead to more effective governance and increase residents’ trust in the local government [40]. Following an exhaustive examination of refereed scholarly databases, the researchers has determined that no past research has explored the indirect influence of public participation on residents’ trust in city administration through the lens of perceived government responsiveness within a unified, comprehensive framework. This study proposes the following hypothesis to address this lacuna in public administration scholarship.

###  H4

*: Perceived local government responsiveness significantly mediates the relationship between perceived public participation and residents’ trust in city administration.*

**Fig 1:**
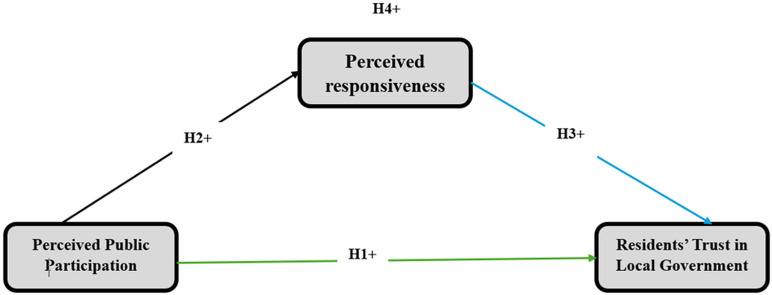
Study model.

The premise of this study is that public participation enhances residents’ trust in city administration by fostering the responsiveness of local authorities. When residents actively engage in local government processes, such as attending public meetings and contributing to decision making, they provide valuable feedback that can lead to more responsive and effective governance. This active participation enhances the perception of increased responsiveness as residents notice that their input is valued and implemented. The perceived responsiveness of local governments mediates the linkage between perceived public participation and residents’ trust in them. Fundamentally, when residents perceive tangible improvements and effective governance resulting from their participation, their perceptions of the city government’s responsiveness also improve, which, in turn, strengthens their trust in the city authority. This study’s conceptual model was constructed to fill the void in the literature on public administration scholarship in Somali settings (see Fig 1).

### Somali context

After gaining independence in 1960, Somalia entered a period of democratic governance with popular votes electing both national and local authorities. Decentralization typified the administrative structure of the nation under elected governments that took office from 1960 to 1969, with democratically elected local councils supervising districts and local administrations nationwide [44]. However, Mohamed Siad Barre’s 1969 military coup marked a significant shift in governance. The military dissolved all elected local government bodies and district councils, effectively centralizing power. Despite this centralization, the military regime established a governmental framework aimed at instilling patriotism, nationalism, and a sense of national identity. However, it did not empower local communities to manage their own affairs.

However, in 2012, Somalia adopted a federal system of government and a provisional federal constitution that clearly stipulates three levels of governance at which citizens have the right to elect both their local and national leaders. However, the Somali national and state governments have not yet delegated authority [13]. This is remarkably true in the local authority in Mogadishu city administration, where citizens have limited control over city operations and administration [45]. The federal president of Somalia nominates the mayor and governor of the Mogadishu local government, and his deputies, based on a 4.5 power-sharing formula. The mayor then appoints district commissioners responsible for running the local district administration. Abdi and Dirie conducted a qualitative study on community participation in decision making in the Mogadishu local government [44]. They reported that residents’ participation in local government affairs is too limited and weak because of many factors that prevent them from involving local government in decision-making processes, such as community distrust in local government, insecurity, deficiency in community engagement skills within the local authority officials, and low skills of local authority leadership. The Heritage Institute for Policy Studies (HIPS) pinpointed several obstacles to meaningful citizen engagement by local government in Somalia, including constitutional vagueness, clan power-sharing, ambiguous political responsibilities and roles, the conduct of government actors, and the manipulation of federal and state governments [13]. Furthermore, the status of the local government in Mogadishu, Somalia’s capital, must be specified under the country’s provisional federal constitution.

The centralized practices in question have eroded residents’ trust in the city administration. Federal presidents’ appointment of city administration leaders has made them more accountable and responsive to federal authority than their constituents. Lack of responsiveness to community needs is a major problem. Structural and political factors exacerbate this situation, including federal government interventions, the ambiguity of the constitution, and clan-based power-sharing arrangements. These factors have hindered the establishment of responsible, responsive, and transparent local governments. Meaningful decentralization and public participation are essential for enhancing legitimacy and trust in local governments and improving their perceptions of responsiveness.

## Methods

### Design, participants, and procedures

This study employed a quantitative online survey design to collect data from the inhabitants of Mogadishu, the capital city and largest city. The city is divided into four main divisions, each subdivided into districts. The following table shows the distribution of divisions and districts in Mogadishu’s local government.

The divisions are the East (comprising the districts of Shibis, Huriwaa, Yaqshid, and Kaaraan), Central division (encompassing Xamarweyne, Wardhiigley, Cabdicasiis, Shangani, and Boondheere districts), West (including Dayniile, Wadajir, Kaxda, and Dharkeyney), and Waliyow Adde (consisting of Waabari, Xamarjajab, Hodan, and Howlwadaag)(See Table 1). Each district of Mogadishu has its own administrative unit headed by a district commissioner appointed by the governor/mayor of Mogadishu. Although no official government census is available, the city’s population is estimated to be roughly 3.5 million. We collected survey data from diverse inhabitants of the four main divisions of Mogadishu City (Eastern, Western, Central, and Waliyow Adde) between January 20^th^ and April 30^th^, 2024. Before starting data data-gathering process, the principal researcher wrote a formal letter to the Ethics Committee of the Faculty of Economics and Management at the Somali National University to seek ethical permission before starting data collection. The committee subsequently granted ethical approval at Somali National University in Mogadishu, Somalia (Approval Number: ECFEMS0002). The researchers distributed an electronic survey administered via Google Forms to gather the data. The researchers marked all the questions as required in Google Forms to reduce the incidence of missing data. The online survey included a cover letter outlining the study’s objectives, clarifying that filling out this survey was voluntary and guaranteeing that refusal to participate would not result in any negative consequences. The researchers obtained written informed consent from each participant by asking them to confirm their agreement to participate in this study before completing the online survey. Moreover, the researchers affirmed that their responses were confidential and kept identities anonymous.

**Table 1:** Administrative divisions and corresponding districts in mogadishu local government.

Division	Districts
Eastern	Shibis
	Huriwaa
	Yaqshid
	Kaaraan
Central	Xamarweyne
	Wardhiigley
	Cabdicasiis
	Shangani
	Boondheere
West	Dayniile
	Wadajir
	Kaxda
	Dharkeyney
Waliyow Adde	Waabari
	Xamarjajab
	Hodan
	Howlwadaag

To establish the necessary sample size for this study, we used an online sample size calculator for structural equation modeling (SEM) developed by Soper [46]. Based on an expected effect size of 0.13, statistical power of 0.80, three latent variables, and 14 observable variables with a p-value of 0.05, the minimum recommended sample size was 734. Our study used a sample size of 800 participants, which exceeded this requirement. Therefore, a total of 800 questionnaires were disseminated via digital media networks, such as Telegram, WhatsApp, Facebook, and email. The researchers sent a reminder message to the participants to encourage participation after a two-week interval, using a convenient sampling technique. The researchers trained university students to help them disseminate the survey questions after they understood the study’s objectives. A total of 446 completed surveys were returned, with an answer rate of 55.6%. In social science research, responses within 30% to 70% are deemed acceptable [47]. After examining the returned questionnaires, the researchers found no missing cases or outliers. Therefore, the researchers used 446 responses that were included in the final analysis. The study subjects’ age range was 18–62, with an average age of about 28. Regarding the participants’ sex, 78.50% were men, and 21.50% were women. Considering the participants’ geographical residence, 37.90% resided in the Eastern division, 22.00% in the Western division, 17.90% in the Waliyow Adde division, and 22.20% in the Central division., 59.0% of the respondents held an undergraduate degree, 37.70% achieved a master’s qualification level, 1.10% completed secondary education, and 2.20% had a PhD.

### Instruments

This study examined three constructs: the endogenous construct of residents’ trust in city government (RTLG), the exogenous construct of perceived public participation (PPP), and the mediating construct of perceived responsiveness (PRP). The study’s model assumes that residents’ trust in city governments is shaped by both perceived public participation and perceived responsiveness (see Fig 1). All latent variables in this study were assessed using a 5-point Likert rating scale, where one corresponds to “strongly disagree” and five corresponds to “strongly agree.”

#### Perceived Public Participation (PPP).

**PPP** was gauged using a four-question scale created and validated by Wang and Van Wart [22]. These items evaluate the extent to which residents believe they are actively engaged in local government decision-making processes. Sample items include “The local government in Mogadishu actively involves citizens in designing alternative programs” and “The local government in Mogadishu actively involves inhabitants in setting the goals and objectives of the local authorities.”

#### Residents’ Trust in Local Government (RTLG).

**RTLG** was assessed with a five-statement scale created and validated by Grimmelikhuijsen [48]. This construct measures the trust inhabitants have in their city authorities, focusing on perceived integrity, benevolence, and competence. Sample items include, “The local government of Mogadishu is acting in the residents’ best interest” and “The local government in Mogadishu is competent.”

#### Perceived Responsiveness (PRP).

To measure the PRP construct, we used the five-item scale created and validated by Vigoda-Gadot and Yuval [49]. These scales capture the extent to which inhabitants believe that the city government is responsive to their demands and concerns. Sample items include “The government responds to residents’ requirements promptly” and “The Mogadishu City administration effectively delivers reliable resolutions to meet residents’ needs.”

## Results

The current study utilized SmartPLS version 4.1.0.8, using covariance-based structural equation Modeling (CB-SEM) to verify the current study’s proposed structural models [50]. Before checking the psychometric standards of the outer (measurement model) and testing the inner (structural) model’s propositions, the researchers checked for potential problems of common method bias (CMB) using SmartPLS version 4.1.0.8 and SPSS 27. After confirming that CMB was not a threat, the study models were analyzed. First, unrotated exploratory factor analysis was performed for each item related to a particular construct. This study showed that this factor explained 47.92% of the average variance, lower than the suggested cutoff of 50%. Moreover, a full collinearity test was used to evaluate the presence of CMB [51]. The study found that all VIF calculated scores for each construct were less than the suggested benchmark of 3.3 [51]. Thus, these results confirm that CMB was not a threat in this study.

### Measurement model

**Fig 2:**
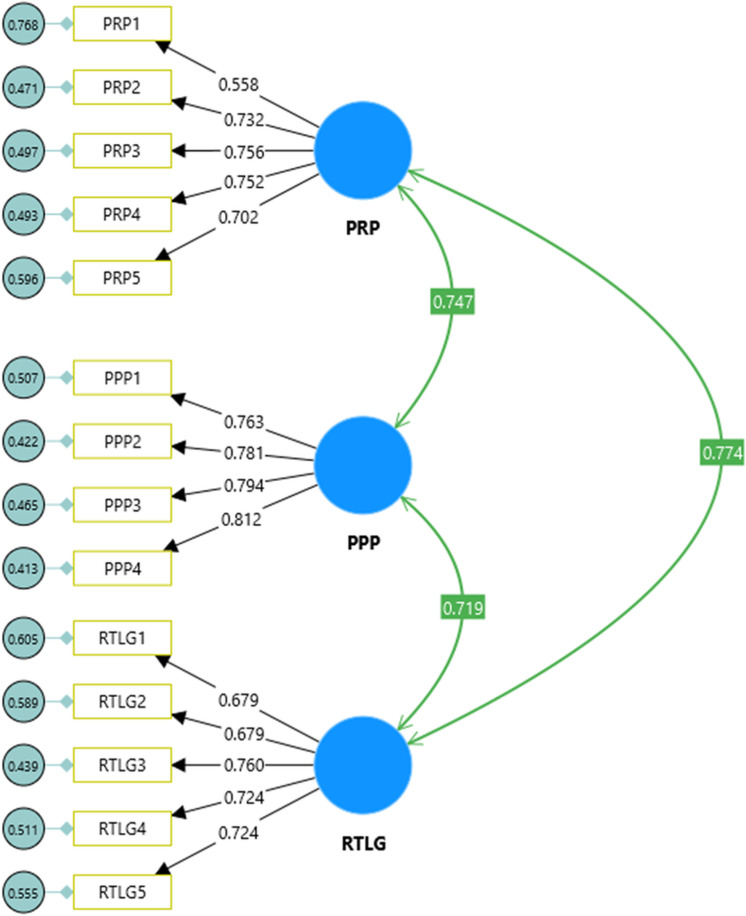
Measurement model.

This current study evaluated the validity and reliability of the measurement model using confirmatory factor analysis (CFA) using CB-SEM with the SmartPLS 4 Software. We selected Covariance-Based Structural Equation Modeling (CB-SEM) because it can manage complex relationships between latent and observed variables, provide comprehensive model fit indices, and rigorously test theoretical models [52]. This was particularly relevant in our study, which involved testing a mediation model. Moreover, CB-SEM’s confirmatory nature and robust analytical capabilities make it a valuable tool for social science researchers seeking to validate theoretical constructs and relationships [52,53]. First, the researchers assessed the factor loadings of the items. The results confirmed a minimum threshold of 0.55, indicating that all indicator loadings were acceptable [54] (Fig 2 and Table 3).

Next, the researchers assessed the study’s overall model fit indices. The computed result (p = 0. 000) revealed that the chi-square test is significant (p < 0.05), indicating that the model may not be a good fit. However, the p-value may be affected by sample size, which can affect the accuracy of the chi-square test when evaluating model fit [37]. Alternatively, the model fit could be assessed using the minimum discrepancy (CMIN/df), which in this study was found to be 1.680 (see Table 2). This study employed the goodness-of-fit index (GFI), comparative fit index (CFI), Tucker-Lewis index (TLI), normed fit index (NFI), standardized root mean square residual (SRMR), and root mean square error of approximation (RMSEA) as model fit indices. Lower values on these indices indicate better model fit. The researchers computed GFI, CFI, TLI, and NFI, obtaining values of 0.961, 0.983, 0.979, and 0.959, respectively. These values exceeded the commonly accepted threshold of 0.90 [55–57], suggesting that the model fits the data well. Finally, the result showed that the SRMR score was 0.031, which is lower than the benchmark of 0.05 [58,59]. The RMSEA value was 0.039, much lower than the recommended cut-off of 0.07 [60], indicating that the current model accurately reflects the data (see Table 2).

**Table 2:** Model fit indices.

Fit Index	Benchmark	Source	Value	Model fit
P	>0.05	[62]	0.000	No
CMIN/df = χ^2^/df	≤3	[37]	1.680	Yes
GFI	>0.90	[55]	0.961	Yes
CFI	>0.90	[57]	0.983	Yes
TLI	>0.90	[56]	0.979	Yes
NFI	>0.90	[55]	0.959	Yes
SRMR	<0.05	[59]	0.031	Yes
RMSEA	<0.07	[60]	0.039	Yes

**Table 3:** Construct validity, reliability, and collinearity.

Latent variable	Indicators	Factor loadings	CR	CA	AVE	VIF
**Perceived Public Participation (PPP)**	PPP1	0.763				1.996
	PPP2	0.781				2.113
	PPP3	0.794	0.867	0.867	0.621	2.129
	PPP4	0.812				2.206
**Perceived Responsiveness (PRP)**	PRP1	0.558				1.325
	PRP2	0.732				1.756
	PRP3	0.756	0.825	0.829	0.495	1.884
	PRP4	0.752				1.935
	PRP5	0.702				1.687
	RTLG1	0.679				1.600
**Residents’ Trust in Local** **Government (RTLG)**	RTLG2	0.679				1.653
	RTLG3	0.760				1.883
	RTLG4	0.724	0.838	0.838	0.509	1.792
	RTLG5	0.724				1.739

Third, the researchers assessed the latent variable reliability of the study model by using composite reliability (CR) and Cronbach’s Alpha (CA), both of which were lower than the required criterion of 0.70 [54]. The average variance extracted (AVE) was used to compute the convergent validity of the study’s constructs. The findings indicated that all scores met the required criterion [54], with the exception of the PRP factor, which exhibited a score of 0.495. However, Fornell and Larcker suggest that convergent validity is endorsed when the CR is higher than 0.80 and the AVE values are less than 0.50 [61]. Finally, the discriminant validity of the model was assessed using the heterotrait-monotrait ratio (HTMT) criterion. The result showed that all HTMT scores met the minimum benchmark of 0.85 [50], signaling that the model exhibited discriminant validity (see Table 4).

**Table 4:** Discriminant validity: (HTMT) criterion.

Latent variable	PPP	PRP	RTLG
Perceived Public Participation (PPP)	**–**		
Perceived Responsiveness (PRP)	0.758	–	
Residents’ Trust in Local Government (RTLG)	0.719	0.790	–

### Examination of the structural model

The researchers must check the model fit indices before ascertaining the proposed hypothesized relationship among study constructs using CB-SEM in SmartPLS4. First, the study’s model fit indices were assessed. An acceptable model fit is met when the CMIN/df values are ≤ 3 [37], GFI > 0.90 [55], CFI > 0.90 [57], TLI > 0.90 [56], NFI > 0.90 [55], RMSEA < 0.05 [60], SRMR < 0.06 [59]. The model fit indices in Table 1 satisfy the recommended minimum threshold of all the indices used in this study: CMIN/df = 1.680, GFI = 0.961, CFI = 0.983, TLI = 0.979, NFI = 0.959, SRMR = 0.031, and RMSEA = 0.039. Thus, the findings reveal that the model fits the proposed relationship quite well (see Table 2). The study’s structural model explained 55.9% of the total variation in perceived responsiveness and 64.4% of the total change in residents’ trust in the local government (see Table 5).

**Table 5:** Model’s Explanatory Power.

Endogenous construct	R^2^
Perceived Responsiveness (PRP)	0.559
Residents’ Trust in Local Government (RTLG)	0.644

### Testing direct relationship

**Fig 3:**
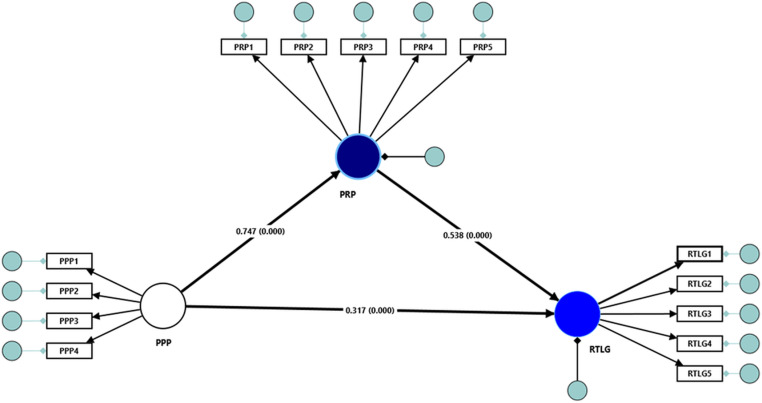
Structural model.

Fig 3 and Table 6 results indicate the significant and positive relationship between perceived public participation (PPP) and residents’ trust in local government (RTLG) (β = 0.317, t = 3.788, p < 0.001). Thus, H1 is empirically supported. This finding suggests that city governments should enhance public participation mechanisms to strengthen trust among residents in local city administration. Similarly, perceived public participation (PPP) was significantly and positively associated with perceived responsiveness (PRP) (β = 0.747, t = 21.101, p < 0.001). Hence, H2 is empirically supported. This result indicates that when local residents perceive higher levels of public participation (PPP), they are more likely to view the local government as responsive (PRP) to their needs. Moreover, the direct effect of perceived responsiveness (PRP) on residents’ trust in local government (RTLG) was significant and positive (β = 0.538, t = 6.310, p < 0.001). Thus, H3 is supported (see Fig 3 and Table 6). This result implies that when local residents perceive that the government is attentive, responsive, and reacts appropriately to their concerns, their trust in local authorities increases.

**Table 6:** Hypotheses testing.

Hypothesized Path	β	t	p	Remarks
H1: PPP→RTLG	0.317	3.788	0.000	√
H2: PPP → PRP	0.747	21.101	0.000	√
H3: PRP→RTLG	0.538	6.310	0.000	√
**Mediation effect**				
H4: PPP → PRP→RTLG	0.402	6.114	0.000	√

### Testing indirect effect (mediation)

A mediation analysis was conducted to test the mediating role of perceived responsiveness (PRP) in the relationship between perceived public participation (PPP) and residents’ trust in local government (RTLG). The results in Table 6 show that the indirect effect of perceived public participation (PPP) on residents’ trust in local government (RTLG) through perceived responsiveness (PRP) was significant and positive (β = 0.402, t = 6.114, p < 0.001). Thus, H4 is empirically supported. This result indicates that PRP partially mediates the relationship between PPP and RTLG.

### Endogeneity evaluation

To check for endogeneity issues, we used Gaussian copula analysis in SmartPLS4. We added another independent variable to our model: the Gaussian copula term. To determine whether endogeneity is a significant concern in our study, we performed a Gaussian copula test to evaluate the copula coefficients and their significance using bootstrapping (with 10,000 subsamples). A p-value below 0.05 for the copula coefficient would suggest a potential endogeneity issue. However, as shown in Table 7, all Gaussian copula values in our model were found to be insignificant (p > 0.05). This indicates that our model does not suffer from endogeneity, further supporting the robustness of the structural model [63].

**Table 7:** Findings of the Gaussian Copula Method for Endogeneity.

Gaussian Copula path	β	t	p
GC: PPP →RTLG	-0.099	0.819	0.206
GC: PPP → PRP	0.052	0.210	0.417
GC: PRP→RTLG	0.245	1.467	0.071

Note: GC: Gaussian Copula.

## Discussion and conclusion

The results supported all proposed hypotheses of the current study, which revealed that PPP is significantly and positively related to PRP and RTLG. Moreover, PRP is positively and significantly associated with the RTLG. Furthermore, PRP positively and significantly mediates the relationship between PPP and the RTLG. The result related to PPP corroborates past study results (see [9,17]), which reported that perceptions of citizens’ perception of participation are associated with a higher level of residents’ trust in city authority. The result signifies that residents are more likely to trust their city administration when they perceive that they have a voice in its administration. Therefore, local governments seeking to enhance public trust should actively engage residents in local authorities’ decision-making processes. Furthermore, prior research supports the association between perceived public participation and responsiveness [64]. These studies reported a statistically significant positive correlation between public participation and perceptions of local government responsiveness. This finding suggests that residents who perceive themselves as participants in the city authority’s decision-making procedures are more likely to develop enhanced perceptions of local authorities’ responsiveness to their needs, expectations, and requirements. Therefore, if local authorities want to improve residents’ perceptions of responsiveness, they should actively engage them in city government administration and decision-making processes.

Moreover, supporting earlier research findings [9,18], which reported that the perception of responsiveness in local government positively predicts residents’ trust in local government, this study revealed that perceived responsiveness is statistically significant and positively related to residents’ trust in the city administration. This finding signifies that when residents perceive their local authorities as responsive to their needs and demands, their trust and confidence in the local government increases. Therefore, local governments in Mogadishu should demonstrate responsiveness to residents’ demands and the need to augment their trust in city authorities.

Finally, the study’s findings revealed a mediating effect of perceived responsiveness on the correlation between perceived public participation and residents’ trust in city authority. Past research confirmed that perceived public participation is associated positively and significantly with the perceived responsiveness of city authority [64]. Furthermore, Halvorsen asserted that citizens’ perceptions of government institutions’ responsiveness significantly increased when they viewed their public participation as high-quality [65]. Moreover, past studies have confirmed that perceived responsiveness is positively and significantly related to residents’ trust in city authority [9,18,66]. While prior research has not examined this mediatory role to the author’s knowledge, this study’s premise is that when residents are involved in decision-making processes, they can meaningfully contribute to formulating local government programs, projects, and policies that respond to needs and demands. This, in turn, encourages a high perception of responsiveness among residents, which may lead to high trust in local authorities. In conclusion, this study argues that enhancing public participation and responsiveness in local government promotes residents’ trust in local authorities. This underscores the crucial pathway for improving local governance and boosting cooperation in post-conflict settings, such as Mogadishu and Somalia.

### Theoretical contribution

Although several previous studies have emphasized the importance of perceived public participation (PPP) in enhancing residents’ trust in local authorities (RTLG) [9,17], the mechanism through which PPP influences RTLG is unclear and has been underexplored in Somali settings. Furthermore, the extant literature on local government ignored and overlooked the transmission effect of the perceived responsiveness of local government (PRP) in the relationship between PPP and RTLG. Therefore, to bridge this paucity in literature, this study assessed the mediating effect of PRP on the relationship between PPP and RTLG in Mogadishu Local government, Somalia. Next, we briefly discuss the theoretical and practical implications of the present study’s results.

First, our study’s findings offer novel insights into the public administration scholarship on RTLG by establishing PPP as its predictor and PRP as its consequence. This topic remains underexplored and inadequately investigated in the context of Somalia’s existing local government literature. Second, notwithstanding that existing literature has effectively investigated perceived local government performance, citizen satisfaction, and perceived public service performance (see [16,67,68] as mediating mechanisms between PPP and trust in government agencies, the study of PRP as an underlying mechanism in the relationship between PPP and RTLG has been neglected and overlooked in existing literature. This study offers novel insights by proposing and validating the mediating role of PRP in the link between PPP and RTLG. Finally, the present study’s findings support the applicability of participatory theory, which contends that democratic public participation in governance and decision making strengthens democracy, legitimacy, and public trust in governmental institutions [34]. Local governments have firsthand knowledge of the needs and preferences of the public when they actively involve residents in decision-making processes, such as town hall meetings, participatory budgeting, and public consultations. Consequently, a responsive government responds to public feedback, establishes credibility, and strengthens trust as citizens see that their needs and preferences are taken seriously. Therefore, one of the main factors influencing individuals’ trust in institutions is their perceptions of how the government listens, reacts, and acts, which in turn influences their trust in the local administration.

### Practical implications

The findings of this study underscore the significance of city government authorities in Mogadishu adopting policies and strategies that emphasize public participation and responsiveness to strengthen resident trust. Enhancing citizen engagement in decision-making procedures and demonstrating transparent, timely responses to community needs can foster trust and promote cooperative relationships between residents and city administrations. Such practices can contribute to more effective governance and bolster the legitimacy of local authorities, particularly in Somalia’s post-war setting. This study provides invaluable suggestions for policymakers seeking to improve resident trust and public engagement in local governance frameworks. Local government officials who want to regain resident trust must establish participatory governance systems, including community forums, public meetings, and online feedback channels, allowing inhabitants to shape policy decisions and build trust in local authorities. Moreover, local administrators and service providers must respond to the needs and preferences of local inhabitants by streamlining services, reducing administrative red tape, and increasing transparency, which can significantly enhance the credibility and trust of the local government. Furthermore, it is essential to emphasize citizen-centered governance in leadership training, as this gives officials the ability and competence to interact with communities, tackle issues, and implement responsive policies that help local governments establish trust and legitimacy.

### Limitations and future research directions

This study provides crucial insights into how perceived public participation influences residents’ trust in local governments within the Mogadishu local authority. Furthermore, perceived responsiveness mediates this relationship. However, several constraints of this study are worth acknowledging. First, the study’s sample size (N = 446) was limited by time and budget, possibly affecting the representation of the city’s diverse population. Future research should include a larger sample to better reflect the city’s demographic diversity. Second, this study’s cross-sectional approach has limitations. Therefore, future studies should utilize time series or longitudinal approaches to enhance predictability and causality. Third, this study used perceived responsiveness as a mediator between perceived public participation and residents’ trust in the city authorities. Future studies should consider other intermediary constructs such as citizens’ satisfaction, social cohesion, and perceived fairness. Moreover, future research should consider the moderating influence of perceived corruption on this relationship. Finally, this study collected data from 446 inhabitants of Mogadishu City using an online quantitative survey. Future research should employ mixed or multi-source data collection methods to examine the constructs under investigation comprehensively.

## Supporting information

S1 File Appendix. Measurement scales for survey variables. Contains the complete list of survey items, Likert-scale anchors (1 = Strongly Disagree, 5 = Strongly Agree), and references for the variables Perceived Public Participation (PPP), Perceived Responsiveness (PRP), and Residents’ Trust in Local Government (RTLG).
(DOCX)

S2 File Dataset. Raw survey responses in SPSS format. Anonymized dataset (.sav) used for all statistical analyses in this study.
(SAV)
