# Supplementary material for: Examining the effects of public participation on residents’ trust in local government: The mediating effect of perceived responsiveness
Source: PLoS One. 2025 May 12;20(5):e0323047. doi: 10.1371/journal.pone.0323047 (PMC12068641; doi:10.1371/journal.pone.0323047)
Supplement: S1 File — Measurement scales for survey variables. Contains the complete list of survey items, Likert-scale anchors (1 = Strongly Disagree, 5 = Strongly Agree), and references for the variables Perceived Public Participation (PPP), Perceived Responsiveness (PRP), and Residents’ Trust in Local Government (RTLG). (DOCX) [file pone.0323047.s001.docx]

**Appendix A. Scale used to gauge each variable.**

| **Variables** | **Items Scale** | **References** |
| --- | --- | --- |
|  | **(1 = Strongly Disagree; 5 = Strongly Agree)** |  |
|  | **PPP1:** The local government in Mogadishu actively involves citizens in designing alternative programs. | Wang and Wart [24] |
| **Perceived Public Participation (PPP)** | **PPP2:** The local authority in Mogadishu actively engages residents in assessing program outcomes. |  |
|  | **PPP3:** The local authority in Mogadishu actively engages residents in determining the local budget. |  |
|  | **PPP4:** The local government in Mogadishu actively involves inhabitants in setting the goals and objectives of the local authorities. |  |
|  | **(1 = Strongly Disagree; 5 = Strongly Agree)** |  |
| **Perceived Responsiveness (PRP)** | **PRP1:** The government is sensitive to public opinions |  |
|  | **PRP2:** The government responds to public requests quickly. | Vigoda-Gadot & Yuval [44] |
|  | **PRP3**: The government is making a sincere effort to support those residents who need help |  |
|  | **PRP4:** The Mogadishu City administration effectively delivers reliable resolutions to meet residents’ needs |  |
|  | **PRP5:** The government responds to residents’ requirements promptly |  |
| **Residents’ Trust in Local**  **Government (RTLG)** | **(1 = Strongly Disagree; 5 = Strongly Agree)** |  |
|  | **RTLG1**: The local government of Mogadishu is acting in the residents’ best interest. | Grimmelikhuijsen [43] |
|  | **RTLG2:** The local authority in Mogadishu operates with integrity. |  |
|  | **RTLG3:** The local authority in Mogadishu is sincere in its operations. |  |
|  | **RTLG4:** The local authorities in Mogadishu carry out their responsibilities effectively. |  |
|  | **RTLG5:** The local government in Mogadishu is competent. |  |
